# Contrast-Associated Acute Kidney Injury

**DOI:** 10.3390/jcm11082167

**Published:** 2022-04-13

**Authors:** Alessandro Mandurino-Mirizzi, Andrea Munafò, Gabriele Crimi

**Affiliations:** 1 Division of Cardiology, Fondazione IRCCS Policlinico San Matteo, 27100 Pavia, Italy; ale.mandurinomirizzi@gmail.com (A.M.-M.); andreamuna1993@gmail.com (A.M.); 2 Interventional Cardiology Unit, Cardio-Thoraco Vascular Department (DICATOV), IRCCS Ospedale Policlinico San Martino, 16100 Genova, Italy; 3 IRCCS Italian Cardiovascular Network & Department of Internal Medicine, University of Genova, 16100 Genova, Italy

**Keywords:** acute kidney injury, nephropathy, contrast media

## Abstract

Contrast-associated acute kidney injury (CA-AKI) is an impairment of renal function, which occurs within days of intravascular administration of iodinated contrast media. Taking into account that minimally invasive cardiac interventions are becoming increasingly popular, compared to traditional surgery, given their impact on prognosis and costs, CA-AKI remains a subject of increasing interest for patients and physicians. This review summarizes the epidemiology and risk stratification, diagnostic criteria, pathophysiology and clinical implications of CA-AKI, providing evidence for the most studied preventive strategies.

## 1. Introduction

Contrast-associated acute kidney injury (CA-AKI) is an impairment of renal function, which occurs within days of intravascular administration of iodinated contrast media (CM). CA-AKI adversely affects clinical outcomes, being associated with increased short- and long-term mortality and major adverse cardiac events (MACE) in patients undergoing percutaneous coronary intervention (PCI) [1,2,3]. Taking into account that minimally invasive cardiac interventions are becoming increasingly popular, compared to traditional surgery, given their impact on prognosis and costs, CA-AKI remains a topic of increasing interest to physicians and researchers. Recent data suggest that several patients are precluded from undergoing invasive procedures, due to concerns about precipitating AKI [4]. However, these concerns arise from an overestimation of the real risk of CA-AKI [5,6].

Being able to identify CA-AKI and individuals at risk of developing CA-AKI is crucial to provide the best treatment to the individual patient. This review summarizes the epidemiology and risk stratification, diagnostic criteria, pathophysiology and clinical implications of CA-AKI, providing evidence for the most studied preventive strategies.

## 2. Epidemiology

In the National Cardiovascular Data Registry Cath-PCI, including 985,737 patients undergoing elective and urgent PCI, the incidence of CA-AKI was 7.1%; dialysis was required in 0.3% of cases [7]. However, the incidence of CA-AKI widely varies across studies because of the different clinical settings and the use of different definitions [8].

CA-AKI incidence is affected by patient-related and contrast-related risk factors.

The most important patient-related factor is baseline renal function; the incidence of CA-AKI ranges from 2% in patients with normal renal function to 30–40% in patients with creatinine > 2 mg/dL [9,10,11]. Other risk factors have been associated with CA-AKI, including advanced age, diabetes mellitus, anemia and elevated serum uric acid [12,13]. However, recent data call into question the independent association of these factors with the development of CA-AKI, due to the fact that they are often associated with CKD [11,14]. Importantly, CA-AKI incidence varies by clinical setting, being higher after an emergency than after elective procedures. In a study by Chong et al., CA-AKI occurred in 12% of patients undergoing PCI during ST-elevation myocardial infarction (STEMI), 9.2% of patients with unstable angina (UA)/non-ST-elevation myocardial infarction (NSTEMI), and 4.5% of patients undergoing elective PCI (*p* = 0.0005) [15]. In addition, heart failure, hemodynamic instability and intra-aortic balloon pump use have been shown to be associated with an increased risk of CA-AKI [3].

Among the contrast-related factors, both the quantitative and qualitative characteristics of CM may influence the incidence of CA-AKI. Higher volumes of CM have been associated with a higher incidence of CA-AKI [16]. On the other hand, the risk of CA-AKI also depends on the osmolality and viscosity of CM; high osmolality and viscosity are associated with nephrotoxic potential [17].

A series of risk stratification models, including patient and procedural factors, have been previously derived and validated from data based on large numbers of patients [18,19,20,21,22,23,24] (Table 1). It is important to note that the overall applicability of each score is deeply dependent on the clinical context of the study from which that score is derived. Furthermore, all these models are limited by the inclusion of variables that are not known before the procedure. A systematic review by Silver and colleagues aimed to evaluate the performance and clinical utility of 12 risk scores for CA-AKI, published from 2004 to 2015 [25]. Despite the high accuracy of most of these risk scores, their usability in clinical practice is extremely limited because of the lack of external validation in multicenter studies, an unclear association between stratification in a risk category and clinical decision making, and a lack of easy-to-use electronic risk calculators [25].

A promising prospect in this field, derived from a recently proposed risk model, is the Mehran 2 CA-AKI risk score [26], which was derived from a large, contemporary, real-world cohort of patients undergoing PCI. This risk score only includes eight pre-procedure clinical variables, and it achieved high discriminatory power in both the derivation (C-statistic 0.72) and internal validation (C-statistic 0.84) cohorts.

## 3. Definition and Diagnosis

The most common definition of CA-AKI in clinical trials is an increase in serum creatinine (SCr) of 0.5 mg/dL (44 μmol/L), or a 25% increase from baseline, within 2–5 days of the procedure [27,28,29]. However, several alternative definitions have been proposed to define CA-AKI. The European Renal Best Practice (ERBP) position statement on the Kidney Disease: Improving Global Outcomes (KDIGO) guidelines defined CA-AKI as an increase by ≥50% from baseline within 7 days after CM exposure, or ≥0.3 mg/dL (26.5 μmol/L) from baseline within 48 h after CM exposure [30].

The Contrast-Induced Nephropathy Consensus Working Panel affirms the use of the relative increase in SCr for the definition of CA-AKI [31]. On the other hand, however, an absolute increase in SCr > 0.5 mg/dL has been shown to be associated with a lower incidence of CA-AKI, but with a stronger association with clinical outcomes [32,33].

In most cases, the increase in SCr during CA-AKI occurs within the first 72 h after CM administration, peaking within 3–5 days [28]. Therefore, it is important to follow the SCr values for at least 72 h after contrast exposure [31].

Although SCr is currently the primary tool for assessing renal function, by measuring the glomerular filtration rate (GFR), the use of the change in SCr to define CA-AKI has important limitations. SCr is not a direct marker of damage to tubular epithelial cells or glomerular endothelial cells. Consequently, the increase in SCr values following an alteration in renal function is related to a reduction in its clearance, and this takes several days to take place [34,35]. It must also be considered that SCr does not depend solely on renal function, but also on the rate of production and volume of distribution [34]. Furthermore, using both the absolute and percentage increases in SCr as diagnostic criteria has some disadvantages [35,36]. Using the relative increases over baseline may lead to delayed diagnosis in patients with chronic kidney disease (CKD) [35]. On the other hand, when an absolute definition is used, SCr is not highly sensitive in patients with low baseline levels [36].

Alternative biomarkers have been proposed to improve early diagnosis and aid management. Neutrophil gelatinase-associated lipocalin (NGAL), a member of the lipocalin family, readily excreted and detected in urine, accumulates in the cortical tubules of the human kidney, blood, and urine after nephrotoxic and ischemic injuries, and represents an early and sensitive biomarker for AKI. NGAL levels showed a sensitivity of 77.8% (95% confidence interval (CI) 62.8–88.0%) and a specificity of 96.3% (95% CI 74.4–99.6%), with a median NGAL cut-off value of 100 ng/mL (95% CI 80–100 ng/mL) [37]. However, in a recent study of patients with acute heart failure, neither NGAL at baseline nor peak NGAL were superior to creatinine for predicting worsening renal function [38].

Cystatin C (CysC), a serum protein constantly produced by all nucleated cell types in the body, is freely filtered out by the glomerular membrane, thanks to its low molecular mass, and its blood concentration correlates with GFR. Importantly, CysC measurements can be interpreted from a single sample, because CysC levels are independent of weight, height, muscle mass, age, and sex. In addition, GFR calculated from the CysC measurement showed an improvement of 0.23 (95% CI 0.18–0.28) for death and 0.10 (95% CI 0.00–0.21) for progression to end-stage renal disease (ESRD), compared with GFR calculated using SCr [39]. For these reasons, CysC is approved for use by the U.S. Food and Drug Administration.

Although such new biomarkers have been proposed, creatinine continues to be used to measure GFR, due to its availability, cost, and amount of data in the literature.

## 4. Pathophysiology

**Figure 1:**
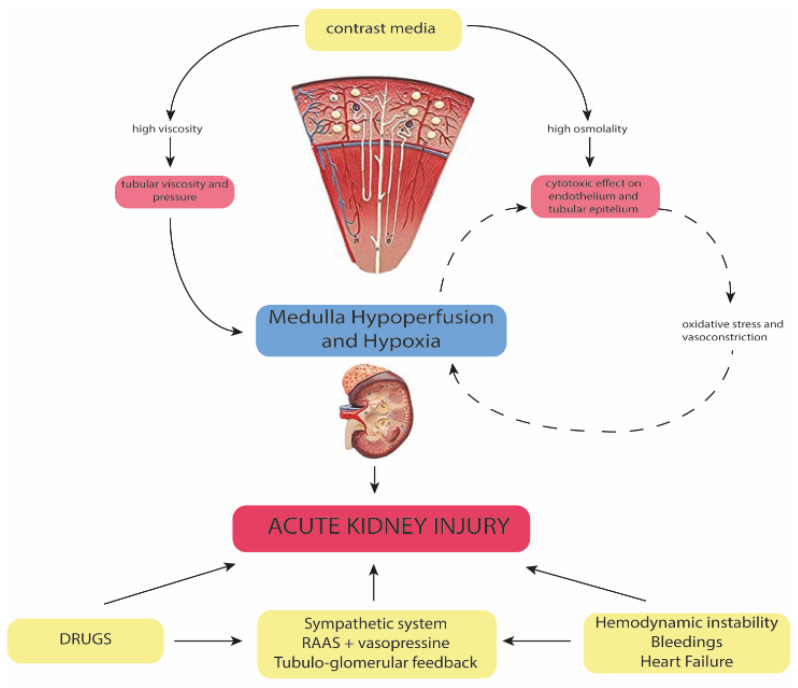
The multifactorial pathophysiology of contrast-associated acute kidney injury. NSAIDs: non-steroidal anti-inflammatory drugs; RAS: renin-angiotensin system; RIC: remote ischemic conditioning; CM: contrast media.

The pathophysiology of CA-AKI is multifactorial, and it is based on a combination of mechanisms. Despite not being completely elucidated, these mechanisms include direct cytotoxic effects, and autocrine, paracrine and endocrine factors, which act on the pre-existing individual risk profile (Figure 1).

Iodinated CM exert a direct cytotoxic effect on epithelial tubular cells, the magnitude of which also depends on the duration of exposure of these cells to CM [28,40,41,42]. This effect has been linked to the ionicity, molecular structure and osmolality of CM. In particular, high-osmolar (HO) CM are associated with high nephrotoxicity. Indeed, compared with low- and iso-osmolar (IO) CM, HOCM are linked with the increased generation of reactive oxygen species (ROS), promoting oxidative stress and renal vasoconstriction [43,44]. The vasoconstriction of descending vasa recta leads to medullary hypoperfusion [45], while the vasoconstrictor effect in the renal cortex results in a reduction in GFR [41]. On the other hand, IOCM are associated with increased viscosity compared to HOCM. High viscosity leads to a reduction in tubular flow velocity, an increase in tubular pressure and an increase in CM retention time [46]. In addition, high viscosity is associated with elevated interstitial and vascular pressures [46], consequently reducing medullary blood flow and promoting hypoperfusion (Figure 1).

Renal hypoperfusion plays a pivotal role in the pathophysiology of CA-AKI [40,47].

In this context, the activation of the sympathetic system, the increased renin–angiotensin–aldosterone activity and the activation of tubular-glomerular feedback can cause relevant renal vasoconstriction, leading to additional renal hypoxic insult [48]. Simultaneously, arginine vasopressin is released and contributes to water retention [48]. Additionally, frequently prescribed drugs, such as non-steroidal anti-inflammatory drugs, antibiotics, angiotensin-converting enzyme inhibitors and angiotensin receptor blockers, may contribute to AKI [49].

Taking into account these considerations, CA-AKI seems to be linked to a set of concomitant causes, rather than to CM alone. Indeed, in animal studies, CM alone has rarely been shown to cause renal damage, unless accompanied by additional damaging agents [50].

## 5. Clinical Implications

CA-AKI has been associated with adverse short- and long-term outcomes, including mortality and cardiovascular events [1,51,52]. The association between CA-AKI and mortality in patients undergoing PCI is profoundly influenced by the clinical setting, being less frequent and less associated with mortality in stable patients than in patients with acute coronary syndrome (ACS) [11,51,53].

CA-AKI is also associated with the progression of CKD. A study including patients with ACS undergoing PCI reported that CA-AKI was an independent predictor for the development of sustained reduction in renal function at 6–8 months (40% in patients with CA-AKI vs. 11% in the control group) [52]. Of note, the authors reported a higher 5-year mortality rate in patients with a sustained reduction in renal function compared to patients with no persistent reduction in renal function (25% vs. 9.4%; *p* = 0.0006) [52]. Indeed, CKD is associated with higher mortality, and the progression to CKD also strongly affects prognosis [2,3].

In many studies reporting an association between CA-AKI and mortality, this association was confounded by baseline clinical characteristics [1], and a discussion arose regarding whether CA-AKI is a marker of an increased risk of adverse outcomes or a mediator of such outcomes. Interestingly, a recent meta-analysis showed that the reduction in AKI incidence failed to reduce the risk of long-term mortality (relative risk 0.97, 95% CI 0.82–1.16) or the development of CKD (relative risk 0.87, 95% CI 0.52–1.46) [54]. Whether a marker or mediator, given the current uncertainty about the causal relationship between CA-AKI and adverse outcomes, it would be important not to exclude patients from invasive procedures just to avoid developing CA-AKI. On the other hand, the reported relationship between CA-AKI and relevant clinical implications demonstrates the need for the implementation of preventive measures of CA-AKI.

## 6. Prevention and Management

The easier preventive measure is to eliminate potentially harmful factors. First, the minimal amount of contrast needed should be used, avoiding HOCM [55]. In addition, all non-essential nephrotoxic medications should be discontinued for 24 h before and 48 h after the procedure [55]. Among potentially nephrotoxic drugs, however, there are only non-convincing data to support the discontinuation of renin–angiotensin system (RAS) inhibitors [56,57,58]. Although intensive RAS inhibition appears to have deleterious effects in hospitalized patients, and probably in the setting of cardiac catheterization and PCI [56,57], the Angiotensin-Converting Enzyme Inhibitor/Angiotensin Receptor Blocker and Contrast-Induced Nephropathy in Patients Receiving Cardiac Catheterization (CAPTAIN) trial failed to demonstrate a difference in CA-AKI occurrence between patients who continued and patients who discontinued RAS inhibitor treatment before coronary angiography (18.4% vs. 10.9%, respectively; hazard ratio (HR): 0.59; 95% CI 0.30–1.19; *p* = 0.16) [58]. Despite these findings, from a pathophysiological point of view, if RAS inhibitors have a chronic beneficial effect on the kidney, by reducing the intraglomerular pressure, in acute disease or after the administration of iodinated CM, this mechanism may be detrimental because of the inhibition of tubulo-glomerular feedback, and the ability to maintain glomerular filtration and a forward flow of urine through the proximal tubules.

The discontinuation of metformin in diabetic patients is recommended, not because this medication increases the risk of CA-AKI, but to avoid the development of lactic acidosis, eventually leading to AKI.

**Figure 2:**
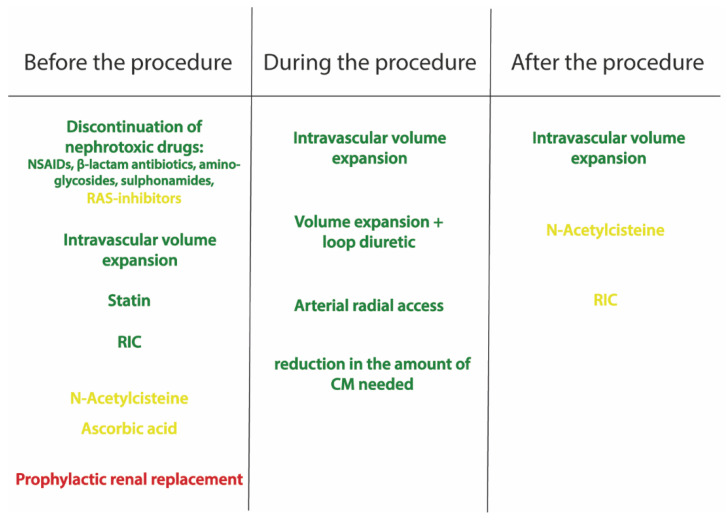
The different strategies proposed for the prevention and treatment of contrast-associated acute kidney injury. The green therapies have been shown to reduce contrast-associated acute kidney injury, the yellow therapies are controversial, while the red treatment is not effective. NSAIDs: non-steroidal anti-inflammatory drugs; RAS: renin–angiotensin system; RIC: remote ischemic conditioning; CM: contrast media.

Several preventive strategies have been proposed (Figure 2).

Intravascular volume expansion plays a pivotal role in the prevention of CA-AKI. It avoids renal hypoperfusion and suppresses the renin–angiotensin–aldosterone system, tubule-glomerular feedback and vasopressin, supporting high urine flow rates and lowering the CM concentration in tubular fluids. Several randomized studies have demonstrated the efficacy of intravenous isotonic saline in reducing CA-AKI [59,60,61].

The current European guidelines recommend hydration with 0.9% sodium chloride at 1–1.5 mL/kg/h for 12 h before the procedure and up to 24 h after the procedure (level of evidence IA) [55].

These recommendations are very nonspecific and often do not fit the heterogeneity of patients presenting during clinical practice. Interestingly, Brar et al. proposed a specific approach for patients undergoing cardiac catheterization. The Prevention of Contrast Renal Injury with Different Hydration Strategies (POSEIDON) trial demonstrated that a strategy of measuring the left ventricular end-diastolic pressure (LVEDP) and expanding the plasma volume was associated with more intensive fluid administration during and after the procedure, and a reduction in CA-AKI, compared with the control group (6.7% vs. 16.3%, relative risk (RR) 0.41, 95% CI 0.22–0.79; *p* = 0.005) [62]. Of note, Qian and colleagues suggested using the right atrial pressure to guide intravascular volume expansion [63].

Multiple randomized trials have compared isotonic bicarbonate solutions to intravenous saline, finding no differences in the rates of renal outcomes [64,65]. More recently, the Prevention of Serious Adverse Events Following Angiography (PRESERVE) trial, with a two-by-two factorial design, randomized patients undergoing non-emergent angiography to receive intravenous isotonic sodium bicarbonate or isotonic saline, as well as oral acetylcysteine or an oral placebo [66]. This trial, prematurely stopped because of futility, showed no significant difference in the incidence of the primary 90-day composite endpoint of death, need for dialysis or persistent impairment in kidney function (4.4% vs. 4.7%, respectively, OR 0.93, 95% CI 0.72–1.22; *p* = 0.63), or in the incidence of CA-AKI (9.5% vs. 8.3%, OR 1.16, 95% CI 0.96–1.41; *p* = 0.13) [66].

The use of loop diuretics is associated with a higher rate of CA-AKI in patients with CKD undergoing PCI [67]. However, volume contraction, imposed by furosemide, may be effective in preventing CA-AKI, if counterbalanced by volume supplementation (level of evidence IIb) [68,69]. By adjusting the rate of intravenous saline infusion, based on urine output, the RenalGuard can provide both volume expansion and valuable diuresis. The suggested intra-procedural urine flow rate is ≥450 mL/h [70]; however, urine output > 150 mL/h before and during the procedure has been shown to significantly reduce the incidence of CA-AKI in patients with CKD [68] and in high-risk patients [68,71].

With regard to pharmacological strategies, although some specific agents, such as N-acetylcysteine, ascorbic acid, aminophylline, trimetazidine, and phenoldopam, have shown benefits in small studies, in large randomized clinical trials, every agent tested to date has failed to prevent or treat CA-AKI [72,73].

Multiple clinical trials have investigated the possible role of N-acetylcysteine (NAC) in the prevention of CA-AKI, relying on its renal vasodilating and antioxidant effects [74]. However, the results of trials and meta-analyses are non-conclusive [75,76,77]. In addition, recently, the PRESERVE trial failed to show a reduction in the rate of CA-AKI in patients treated with acetylcysteine, compared with a placebo (9.1% vs. 8.7%, respectively, OD 1.06, 95% CI 0.87–1.28; *p* = 0.58) [66].

Administering high-dose statins before catheterization has been shown to reduce the incidence of CA-AKI [78]. It has been hypothesized that the nephroprotective action of statins is related to the inhibition of contrast uptake into renal tubular cells, the attenuation of endothelial dysfunction and oxidative stress, anti-inflammation, and antiproliferation of mesangial cells, and the protection of podocytes. The Protective effect of Rosuvastatin and Antiplatelet Therapy On contrast-induced acute kidney injury and myocardial damage in patients with Acute Coronary Syndrome (PRATO-ACS) trial showed that the statin group had a significantly lower rate of CA-AKI than the group without statin (6.7% vs. 15.1%, adjusted OR 0.38, 95% CI 0.20–0.71; *p* = 0.003) [79]. In addition, there was a decrease in 30-day composite death, dialysis, MI, stroke, and persistent renal damage in the statin group (3.6% vs. 7.9%, respectively; *p* = 0.036) [79]. In patients with diabetes and CKD undergoing coronary or peripheral angiography, with or without intervention, rosuvastatin was effective in reducing the incidence of CA-AKI (2.3% vs. 3.9%; *p* = 0.01) [80]. The current European guidelines recommend considering high-dose statin therapy for the prevention of CA-AKI, possibly with pretreatment for naïve patients [55].

In the pathophysiology of CA-AKI, medullary vasoconstriction can cause ischemic/reperfusion injury. Remote ischemic conditioning (RIC), which includes remote ischemic pre-conditioning and remote ischemic post-conditioning, is an intriguing intervention to reduce ischemic/reperfusion injury and improve clinical outcomes [81]. A meta-analysis showed that RIC reduced the incidence of CA-AKI, compared with the control group (OR 0.52, 95% CI 0.34–0.77; *p* = 0.001) [82].

Importantly, among the other suggested practical preventive measures, radial access should be preferred for coronary angiography and interventions, compared with femoral access [83]. In the AKI-MATRIX study, AKI was reduced in the radial access group, compared with the femoral group (15.4% vs. 17.4%, respectively, OR 0.87, 95% CI 0.77–0.98; *p* = 0.018) [83]. Despite the mechanisms not being clear, it could be supposed that these results are linked to the reduction in access-related bleeding events.

## 7. Conclusions

CA-AKI remains a matter of concern for patients undergoing diagnostic and therapeutic procedures that require iodinated contrast administration. To understand the real clinical relevance of the problem, a more precise definition of CA-AKI, possibly using more specific biomarkers, is needed. Future studies should clarify the possible clinical relevance of the toxic effect of CM and the need to develop new prophylactic and therapeutic strategies to improve survival.

## Figures and Tables

**Table 1:** Available risk scores for the prediction of CA-AKI.

Study	Population ^a^	Time Period	N of Variables	Only Pre-Procedural Variables	CA-AKI Definition	C-Statistics
Meharan et al. *JACC* 2004	5571 patients undergoing PCI (only chronic CS)	-	8	No	Increase in SCr ≥ 25% or ≥0.5 mg/dL within 48 h	0.69
Marenzi et al. *JACC* 2004	208 patients undergoing PCI (only acute CS)	2001–2003	5	No	Increase in SCr ≥ 0.5 mg/dL within 72 h	-
Bartholomew et al. *Am. J. Cardiol.* 2004	10,481 (both acute and chronic CS)	1993–1998	8	No	Increase in SCr ≥ 1 mg/dL	0.89
Tziakas et al. *Int. J. Cardiol.* 2011	488 patients undergoing PCI (both acute and chronic CS)	2008–2010	5	No	Increase in SCr ≥ 25% or ≥0.5 mg/dL within 48 h	0.759
Gurm et al. *JACC* 2013	48,001 PCI procedures (both acute and chronic CS)	2010–2012	15	Yes	Increase in SCr ≥ 0.5 mg/dL within 7 days	0.839
Gao et al. *Clin. Exp. Nephrol.* 2014	2764 patients undergoing coronary angiography or PCI (both acute and chronic CS)	2005–2010	7	No	Increase in SCr ≥ 44.2 umol/L or ≥25% and >upper limit of normal value within 72 h	0.76
Tsai et al. *JAHA* 2014	662,504 patients undergoing PCI (both acute and chronic CS)	2009–2011	11	Yes	Increase in SCr ≥ 50% or ≥0.3 mg/dL	0.71
Meharan et al. *Lancet* 2021	14,616 patients undergoing PCI (both acute and chronic CS)	2012–2017	8	Yes	Increase in SCr ≥ 50% or ≥0.3 mg/dL within 48 h	0.72

CA-AKI = contrast-associated acute kidney injury; CS = coronary syndrome; PCI = percutaneous coronary intervention; SCr = serum creatinine. ^a^ refers to the study derivation cohort.

## Data Availability

No new data were created or analyzed in this study. Data sharing is not applicable to this article.

## References

[1] James M.T., Samuel S.M., Manning M.A., Tonelli M., Ghali W.A., Faris P., Knudtson M.L., Pannu N., Hemmelgarn B.R. (2013). Contrast-induced acute kidney injury and risk of adverse clinical outcomes after coronary angiography: A systematic review and meta-analysis. Circ. Cardiovasc. Interv..

[2] Kini A.S., Sarkar K., Rafael O.C., Jakkula M., Kaplish D., Lee P., Suleman J., Krishnan P., Kim M.C., Sharma S.K. (2009). Serum creatinine ratio: A novel predictor of mortality after percutaneous coronary intervention in patients with normal and abnormal renal function. Catheter. Cardiovasc. Interv..

[3] Dangas G., Iakovou I., Nikolsky E., Aymong E.D., Mintz G.S., Kipshidze N.N., Lansky A.J., Moussa I., Stone G.W., Moses J.W. (2005). Contrast-induced nephropathy after percutaneous coronary interventions in relation to chronic kidney disease and hemodynamic variables. Am. J. Cardiol..

[4] McDonald R.J., McDonald J.S., Bida J.P., Carter R.W., Fleming C.J., Misra S., Williamson E.E., Kallmse D.F. (2013). Intravenous contrast material-induced nephropathy: Causal or coincident phenomenon?. Radiology.

[5] Rudnick M.R., Berns J.S., Cohen R.M., Goldfarb S. (1994). Nephrotoxic risks of renal angiography: Contrast media-associated nephrotoxicity and atheroembolism—A critical review. Am. J. Kidney Dis..

[6] Weisbord S.D., Mor M.K., Resnick A.L., Hartwig K.C., Sonel A.F., Fine M.J., Palevsky P.M. (2008). Prevention, incidence, and outcomes of contrast-induced acute kidney injury. Arch. Intern. Med..

[7] Tsai T.T., Patel U.D., Chang T.I., Kennedy K.F., Masoudi F.A., Matheny M.E., Kosiborod M., Amin A.p., Messenger J.C., Rumsfeld J.S. (2014). Contemporary incidence, predictors, and outcomes of acute kidney injury in patients undergoing percutaneous coronary interventions: Insights from the NCDR Cath-PCI Registry. J. Am. Coll. Cardiol. Intv..

[8] Solomon R., Dauerman H.L. (2010). Contrast-induced acute kidney injury. Circulation.

[9] McCullough P.A., Adam A., Becker C.R., Davidson C., Lameire N., Stacul F., Tumlin J., CIN Consensus Working Panel (2006). Epidemiology and prognostic implications of contrast-induced nephropathy. Am. J. Cardiol..

[10] McCullough P.A., Wolyn R., Rocher L.L., Levin R.N., O’Neill W.W. (1997). Acute renal failure after coronary intervention: Incidence, risk factors, and relationship to mortality. Am. J. Med..

[11] Stacul F., van der Molen A.J., Reimer P., Webb J.A.W., Thomsen H.S., Morcos S.K., Almén T., Aspelin P., Bellin M.F., Clement O. (2011). Contrast induced nephropathy: Updated ESUR contrast media safety committee guidelines. Eur. Radiol..

[12] Ohno Y., Maekawa Y., Miyata H., Inoue S., Ishikawa S., Sueyoshi K., Noma S., Kawamura A., Kohsaka S., Fukuda K. (2013). Impact of periprocedural bleeding on incidence of contrast induced acute kidney injury in patients treated with percutaneous coronary intervention. J. Am. Coll. Cardiol..

[13] Mandurino-Mirizzi A., Kajana V., Cornara S., Somaschini A., Demarchi A., Galazzi M., Crimi G., Ferlini M., Camporotondo R., Gnecchi M. (2021). Elevated serum uric acid is a predictor of contrast associated acute kidney injury in patient with ST-segment elevation myocardial infarction undergoing primary percutaneous coronary intervention. Nutr. Met. Cardiovasc. Dis..

[14] Rudnick M.R., Goldfarb S., Wexler L., Ludbrook P.A., Murphy M.J., Halpern E.F., Hill J.A., Winniford M., Cohen M.B., VanFossen D.B. (1995). Nephrotoxicity of ionic and nonionic contrast media in 1196 patients: A randomized trial: The Iohexol Cooperative Study. Kidney Int..

[15] Chong E., Poh K.K., Liang S., Soon C.Y., Tan H.C. (2010). Comparison of risksand clinical predictors of contrast-induced nephropathy in patients undergoing emergency versus nonemergency percutaneous coronaryinterventions. J. Interv. Cardiol..

[16] Owen R.J., Hiremath S., Myers A., Fraser-Hill M., Barrett B.J. (2014). Canadian association of radiologists consensus guidelines for the prevention ofcontrast-induced nephropathy: Update 2012. Can. Assoc. Radiol. J..

[17] Seeliger E., Sendeski M., Rihal C.S., Persson P.B. (2012). Contrast-induced kidney injury: Mechanisms, risk factors, and prevention. Eur. Heart J..

[18] Marenzi G., Lauri G., Assanelli E., Campodonico J., De Metrio M., Marana I., Grazi M., Veglia F., Bartorelli A.L. (2004). Contrast-induced nephropathy in patients undergoing primary angioplasty for acute myocardial infarction. J. Am. Coll. Cardiol..

[19] Mehran R., Aymong E.D., Nikolsky E., Lasic Z., Iakovou I., Fahy M., Mintz G.S., Lansky A.J., Moses J.W., Stone G.W. (2004). A simple riskscore for prediction of contrast-induced nephropathy after percutaneous coronary intervention: Development and initial validation. J. Am. Coll. Cardiol..

[20] Bartholomew B.A., Harjai K.J., Dukkipati S., Boura J.A., Yerkey M.W., Glazier S., Grines C.L., O’Neill W.W. (2004). Impact of nephropathy after percutaneous coronary intervention and a method for risk stratification. Am. J. Cardiol..

[21] Tziakas D., Chalikias G., Stakos D., Apostolakis S., Adina T., Kikas P., Alexoudis A., Passadakis P., Thodis E., Vargemezis V. (2013). Development ofan easily applicable risk score model for contrast-induced nephropathy prediction after percutaneous coronary intervention: A novel approach tailored to current practice. Int. J. Cardiol..

[22] Gao Y.M., Li D., Cheng H., Chen Y.P. (2014). Derivation and validation of a risk score for contrast-induced nephropathy after cardiac catheterization in Chinese patients. Clin. Exp. Nephrol..

[23] Gurm H.S., Seth M., Kooiman J., Share D. (2013). A novel tool for reliable and accurate prediction of renal complications in patients undergoing percutaneous coronary intervention. J. Am. Coll. Cardiol..

[24] Tsai T.T., Patel U.D., Chang T.I., Kennedy K.F., Masoudi F.A., Matheny M.E., Kosiborod M., Amin A.P., Weintraub W.S., Curtis J.P. (2014). Validated contemporary risk model of acute kidney injury in patients undergoing percutaneous coronary interventions: Insights from the National Cardiovascular Data Registry Cath-PCI Registry. J. Am. Heart Assoc..

[25] Silver S.A., Shah P.M., Chertow G.M., Harel S., Wald R., Harel Z. (2015). Risk prediction models for contrast induced nephropathy: Systematic review. BMJ.

[26] Mehran R., Ruth Owen R., Chiarito M., Baber U., Sartori S., Cao D., Nicolas J., Pivato C.A., Nardin M., Krishnan P. (2021). A contemporary simple risk score for prediction of contrast-associated acute kidney injury after percutaneous coronary intervention: Derivation and validation from an observational registry. Lancet.

[27] McCullough P.A. (2008). Radiocontrast-induced acute kidney injury. Nephron Physiol..

[28] McCullough P.A. (2008). Contrast-induced acute kidney injury. J. Am. Coll. Cardiol..

[29] Thomsen H.S. (2003). Guidelines for contrast media from the European Society of Urogenital Radiology. AJR Am. J. Roentgenol..

[30] Fliser D., Laville M., Covic A., Fouque D., Vanholder R., Juillard L., Van Biesen W., Ad-hoc working group of ERBP (2012). A European Renal Best Practice (ERBP) position statement on the Kidney Disease Improving Global Outcomes (KDIGO) clinical practice guidelines on acute kidney injury: Part 1, Definitions, conservative management and contrast-induced nephropathy. Nephrol. Dial. Transplant..

[31] Solomon R., Deray G. (2006). How to prevent contrast-induced nephropathy and manage risk patients: Practical recommendations. Kidney Int. Suppl..

[32] Slocum N.K., Grossman P.M., Moscucci M., Smith D.S., Aronow H.D., Dixon S.R., Share D., Gurm H.S. (2012). The changing definition of contrast induced nephropathy and its clinical implications: Insights from the Blue Cross Blue Shield of Michigan Cardiovascular Consortium (BMC2). Am. Heart J..

[33] Budano C., Levis M., D’Amico M., Usmiani T., Fava A., Sbarra P., Burdese M., Segoloni G.P., Colombo A., Marra S. (2011). Impact of contrast-induced acute kidney injury definition on clinical outcomes. Am. Heart J..

[34] Moran S.M., Myers B.D. (1985). Course of acute renal failure studied by a model of creatinine kinetics. Kidney Int..

[35] Waikar S.S., Bonventre J.V. (2009). Creatinine kinetics and the definition of acute kidney injury. J. Am. Soc. Nephrol..

[36] Lin J., Fernandez H., Shashaty M.G., Negoianu D., Testani J.M., Berns J.S., Parikh C.R., Wilson F.P. (2015). False-positive rate of AKI using consensus creatinine-based criteria. Clin. J. Am. Soc. Nephrol..

[37] Haase M., Bellomo R., Devarajan P., Schlattmann P., Haase-Fielitz A., NGAL Meta-analysis Investigator Group (2009). Accuracy of neutrophil gelatinase-associated lipocalin (NGAL) in diagnosis and prognosis in acute kidney injury: A systematic review and meta-analysis. Am. J. Kidney Dis..

[38] Maisel A.S., Wettersen N., van Veldhuisen D.J., Mueller C., Filippatos G., Nowak R., Hogan C., Kontos M.C., Cannon C.M., Muller G.A. (2016). Neutrophil gelatinase-associated lipocalin for acute kidney injury during acute heart failure hospitalizations: The AKINESIS study. J. Am. Coll. Cardiol..

[39] Shlipak M.G., Matsushita K., Arnlov J., Inker L.A., Katz R., Polkinghorne K.R., Rothenbacher D., Sarnak M.J., Astor B.C., Coresh J. (2013). Cystatin Cversus creatinine in determining risk based on kidney function. N. Engl. J. Med..

[40] Sendeski M.M. (2011). The pathophysiology of renal tissue damage by iodinated contrast media. Clin. Exp. Pharmacol. Physiol..

[41] Thomsen H.S., Morcos S.K., Barrett B.J. (2008). Contrast-induced nephropathy: The wheel has turned 360 degrees. Acta Radiol..

[42] Hardiek K., Katholi R.E., Ramkumar V., Deitrick C. (2001). Proximal tubule cell response to radiographic contrast media. Am. J. Physiol. Renal Physiol..

[43] Katholi R.E., Taylor G.J., McCann W.P., Wookds W.T., Womack K.A., McCoy C.D., Katholi C.R., Moses H.W., Mishkel G.J., Lucore C.L. (1995). Nephrotoxicity from contrast media: Attenuation with theophylline. Radiology.

[44] Schnackenberg C.G. (2002). Physiological and pathophysiological roles of oxygen radicals in the renal microvasculature. Am. J. Physiol. Regul. Integr. Comp. Physiol..

[45] Sendeski M., Patzak A., Pallone T.L., Cao C., Persson A.E., Persson P.B. (2009). Iodixanol, constriction of medullary descending vasa recta, and risk for contrast medium-induced nephropathy. Radiology.

[46] Seeliger E., Flemming B., Wronski T., Ladwig M., Arakelyan K., Godes M., Mockel M., Persson P.B. (2007). Viscosity of contrast media perturbs renal hemodynamics. J. Am. Soc. Nephrol..

[47] Bartorelli A.L., Marenzi G. (2008). Contrast-induced nephropathy. J. Interv. Cardiol..

[48] Schrier R.W., Wang W. (2004). Acute renal failure and sepsis. N. Engl. J. Med..

[49] Bentley M.L., Corwin H.L., Dasta J. (2010). Drug-induced acute kidney injury in the critically ill adult: Recognition and prevention strategies. Crit. Care Med..

[50] Kiss N., Hamar P. (2016). Histopathological evaluation of contrast-induced acute kidney injury rodent models. Biomed Res. Int..

[51] Crimi G., Leonardi S., Costa F., Ariotti S., Tebaldi M., Biscaglia S., Valgimigli M. (2015). Incidence, prognostic impact, and optimal definition of contrast-induced acute kidney injury in consecutive patients with stable or unstable coronary artery disease undergoing percutaneous coronary intervention insights from the all-comer PRODIGY trial. Catheter. Cardiovasc. Interv..

[52] Nemoto N., Iwasaki M., Nakanishi M., Araki T., Utsunomiya M., Hori M., Ikeda N., Makino K., Itaya H., Iijima R. (2014). Impact of continuous deterioration of kidney function 6 to 8 months after percutaneous coronary intervention for acute coronary syndrome. Am. J. Cardiol..

[53] Wickenbrock I., Perings C., Maagh P., Quack I., Van Bracht M., Prull M.W., Plehn G., Trappe H.J., Meissner A. (2009). Contrast medium induced nephropathy in patients undergoing percutaneous coronary intervention for acute coronary syndrome: Differences in STEMI and NSTEMI. Clin. Res. Cardiol..

[54] Coca S.G., Zabetian A., Ferket B.S., Zhou J., Testani J.M., Garg A.X., Parikh C.R. (2016). Evaluation of short-term changes in serum creatinine level as a meaningful end point in randomized clinical trials. J. Am. Soc. Nephrol..

[55] Neumann F.J., Sousa U.V.A.M., Ahlsson A., Alfonso F., Banning A.P., Benedetto U., Byrne R.A., Collet J.P., Falk V., Head S.J. (2019). 2018 ESC/EACTS Guidelines on myocardial revascularization: The task force on myocardial revascularization of the European Society of Cardiology (ESC) and the European Association for Cardio-Thoracic Surgery (EACTS) developed with the special contribution of the European Association of Percutaneous Cardiovascular Interventions (EAPCI). Eur. Heart J..

[56] Fried L.F., Duckworth W., Zhang J.H., O’Connor T., Brophy M., Emanuele N., Huang G.D., McCullough P.A., Palevsky P.A., Seliger S. (2009). Design of combination angiotensin receptor blocker and angiotensin converting enzyme inhibitor for treatment of diabetic nephropathy (VA NEPHRON-D). Clin. J. Am. Soc. Nephrol..

[57] Parving H.H., Brenner B.M., McMurray J.J., De Zeeuw D., Haffner S.M., Solomon S.D., Chaturvedi N., Persson F., Desai A.S., Nicolaides M. (2012). Cardiorenal end points in a trial of aliskiren for type 2 diabetes. N. Engl. J. Med..

[58] Bainey K.R., Rahim S., Etherington K., Rokoss M.L., Natarajan M.K., Velianou J.L., Brons S., Mehta S.R., CAPTAIN Investigators (2015). Effects of withdrawing vs continuing renin-angiotensin blockers on incidence of acute kidney injury in patients with renal insufficiency undergoing cardiac catheterization: Results from the angiotensin converting enzyme inhibitor/angiotensin receptor blocker and contrast induced nephropathy in patients receiving cardiac catheterization (CAPTAIN) trial. Am. Heart J..

[59] Solomon R., Werner C., Mann D., D’Elia J., Silva P. (1994). Effects of saline, mannitol, and furosemide to prevent acute decreases in renal function induced by radiocontrast agents. N. Engl. J. Med..

[60] Trivedi H.S., Moore H., Nasr S., Aggarwal K., Agrawal A., Goel P., Hewett J. (2003). A randomized prospective trial to assess the role of saline hydration on the development of contrast nephrotoxicity. Nephron. Clin. Pract..

[61] Mueller C., Buerkle G., Buettner H.J., Petersen J., Perruchoud A.P., Eriksson U., Marsch S., Roskamm H. (2002). Prevention of contrast media-associated nephropathy: Randomized comparison of 2 hydration regimens in 1620 patients undergoing coronary angioplasty. Arch. Intern. Med..

[62] Brar S.S., Aharonian V., Mansukhani P., Moore N., Shen A.Y.J., Jorgensen M., Dua A., Short L., Kane K. (2014). Haemodynamic-guided fluid administration for the prevention of contrast-induced acute kidney injury: The POSEIDON randomized controlled trial. Lancet.

[63] Qian G., Fu Z., Guo J., Cao F., Chen Y. (2016). Prevention of contrast-induced nephropathy by central venous pressure-guided fluid administration in chronic kidney disease and congestive heart failure patients. JACC Cardiovasc. Interv..

[64] Solomon R., Gordon P., Manoukian S.V., Abbott J.D., Kereiakes D.J., Jeremias A., Kim M., Dauerman H.L., BOSS Trial Invetigators (2015). Randomized trial of bicarbonate or saline study for the prevention of contrast-induced nephropathy in patients with CKD. Clin. J. Am. Soc. Nephrol..

[65] Brar S.S., Shen A.Y., Jorgensen M.B., Kotlewski A., Aharonian V.J., Desai N., Ree M., Shah A.I., Burchette R.J. (2008). Sodium bicarbonate vs. sodium chloride for the prevention of contrast medium-induced nephropathy in patients undergoing coronary angiography: A randomized trial. JAMA.

[66] Weisbord S.D., Gallagher M., Jneid H., Garcia S., Cass A., Thwin S.S., Connor T.A., Chertow G.M., Bhatt D.L., Shunk K. (2018). Outcomes after angiography with sodium bicarbonate and acetylcysteine. N. Engl. J. Med..

[67] Weisbord S.D., Palevsky P.M. (2010). Strategies for the prevention of contrast-induced acute kidney injury. Curr. Opin. Nephrol. Hypertens..

[68] Briguori C., Visconti G., Focaccio A., Airoldi F., Valgimigli M., Sangiorgi G.M., Golia B., Ricciardelli B., Condorelli G., REMEDIAL II Investigators (2011). Renal insufficiency after contrast media administration trial ii (remedial ii): Renal guard system in high-risk patients for contrast-induced acute kidney injury. Circulation.

[69] Marenzi G., Ferrari C., Marana I., Assanelli E., De Metrio M., Teruzzi G., Veglia F., Fabbiocchi F., Montorsi P., Bartorelli A.L. (2012). Prevention of contrast nephropathy with furosemide-induced diuresis and matched hydration—The MYTHOS trial. JACC Cardiovasc. Interv..

[70] Briguori C., Visconti G., Donahue M., De Micco F., Focaccio A., Golia B., Signoriello G., Ciardiello C., Donnarumma E., Condorelli G. (2016). Renal Guard system in high-risk patients for contrast-induced acute kidney injury. Am. Heart J..

[71] Solomon R. (2014). Forced diuresis with the Renal-Guard system: Impact on contrast induced acute kidney injury. J. Cardiol..

[72] ACT-Investigators (2011). Acetylcysteine for prevention of renal outcomes in patients undergoing coronary and peripheral vascular angiography: Main results from the randomized Acetylcysteine for Contrast-induced nephropathy Trial (ACT). Circulation.

[73] Sadat U., Usman A., Gillard J.H., Boyle J.R. (2013). Does ascorbic acid protect against contrast-induced acute kidney injury in patients undergoing coronary angiography: A systematic review with meta-analysis of randomized, controlled trials. J. Am. Coll. Cardiol..

[74] DiMari J., Megyesi J., Udvarbelyi N., Price P., Davis R., Safirstein R. (1997). N-acetylcysteine ameliorates ischemic renal failure. Am. J. Physiol..

[75] Sun Z., Fu Q., Cao J., Jin W., Cheng L., Li Z. (2013). Intravenous N-acetylcysteine for prevention of contrast-induced nephropathy: A meta-analysis of randomized, controlled trials. PLoS ONE.

[76] Xu R., Tao A., Bai Y., Deng Y., Chen G. (2016). Effectiveness of n-acetylcysteine for the prevention of contrast-induced nephropathy: A systematic review and meta-analysis of randomized controlled trials. J. Am. Heart Assoc..

[77] Guo Z., Liu J., Lei L., Xue Y., Liu L., Huang H., Chen S., Liu Y., Lin Y., Tao J. (2020). Effect of N-acetylcysteine on prevention of contrast-associated acute kidney injury in patients with STEMI undergoing primary percutaneous coronary intervention: A systematic review and meta-analysis of randomized controlled trials. BMJ Open.

[78] Li Y., Liu Y., Fu L., Mei C., Dai B. (2012). Efficacy of short-term high-dose statin in preventing contrast-induced nephropathy: A meta-analysis of seven randomized controlled trials. PLoS ONE.

[79] Leoncini M., Toso A., Maioli M., Tropeano F., Villani S., Bellandi F. (2014). Early high-dose rosuvastatin for contrast-induced nephropathy prevention in acute coronary syndrome: Results from the PRATO-ACS Study (Protective Effect of Rosuvastatin and Antiplatelet Therapy On contrast-induced acute kidney injury and myocardial damage in patients with Acute Coronary Syndrome). J. Am. Coll. Cardiol..

[80] Han Y., Zhu G., Han L., Hou F., Huang W., Liu H., Gan J., Jiang T., Li X., Wang W. (2014). Short-term rosuvastatin therapy for prevention of contrast induced acute kidney injury in patients with diabetes and chronic kidney disease. J. Am. Coll. Cardiol..

[81] Crimi G., Ferlini M., Gallo F., Sormani M.P., Raineri C., Bramucci E., De Ferrari G.M., Pica S., Marinoni B., Repetto A. (2014). Remote ischemic postconditioning as a strategy to reduce acute kidney injury during primary PCI_ a post-hoc analysis of a randomized trial. Int. J. Cardiol..

[82] Bei W.J., Duan C.Y., Chen J.Y., Wang K., Liu Y.H., Liu Y., Tan N. (2016). Remote ischemic conditioning for preventing contrast-induced acute kidney injury in patients undergoing percutaneous coronary interventions/coronary angiography: A meta-analysis of randomized controlled trials. J. Cardiovasc. Pharmacol. Ther..

[83] Andò G., Cortese B., Russo F., Rothenbuhler M., Frigoli E., Gargiulo G., Briguori C., Vranckx P., Leonardi S., Guiducci V. (2017). Acute kidney injury after Radial or Femoral Access for Invasive Acute Coronary Syndrome Management: AKI-MATRIX. J. Am. Coll. Cardiol..

